# Reflecting real-world patients with mesothelioma in research: an interim report of baseline characteristics from the ASSESS-meso cohort

**DOI:** 10.1183/23120541.00467-2023

**Published:** 2023-12-27

**Authors:** Ruairi J.H. Conway, Natalie Smith, William Cooper, Geraldine Lynch, Sonia Patole, Jenny Symonds, Anthony Edey, Nick A. Maskell, Anna C. Bibby, Natalie Zahan-Evans, Emma Tucker, Louise Stadon, Nick Maskell, John Harvey, Helen Day, Najib Rahman, Janet Fallon, Dawn Redwood, Tania Wainwright, Sarah Morgan, Liz Darlison, James Walter, Vidan Masani, John Corcoran, Mairead Dixon, Kate Slaven, Avinash Aujayeb, Leah Taylor, Lorell Dismore, Louise Brown, Paul Beckett, Kevin Blyth, Selina Tsim, John Maclay, Alexandra Macpherson, Giles Cox, Paul Shaw, Naomi Chamberlin, Malcolm Lawson, Dipak Mukherjee, Gairin Dancey, Rafiqul Islam, Eleanor Mishra, Tony Pope, Henry Steer, Oliver Bintcliffe, Duneesha de Fonseka

**Affiliations:** 1 Department of Respiratory Medicine, North Bristol NHS Trust, Bristol, UK; 2 Academic Respiratory Unit, School of Translational Sciences, University of Bristol, Bristol, UK; 3 Department of Radiology, North Bristol NHS Trust, Bristol, UK; 4 For a list of the members of the ASSESS-meso Collaborative Group see the Acknowledgements

## Abstract

**Objective:**

Mesothelioma varies in clinical phenotype and survival. Clinical trials are unavoidably affected by selection bias, reducing generalisability. ASSESS-meso is a UK, multicentre, prospective, mesothelioma cohort study (ISRCTN61861764). This pre-specified interim analysis, conducted when recruitment reached 25% of target, summarised participant characteristics and evaluated external validity through comparison with real-world and clinical trial cohorts.

**Methods:**

The study took place at 14 hospitals across the UK. People diagnosed with mesothelioma, at any anatomical site, were eligible. Clinical, radiological and biochemical data were collected at enrolment. In this interim report, the external validity of the cohort was investigated through comparison of baseline demographic data with populations included in the 2020 UK National Mesothelioma Audit (real-world cohort), and CHECKMATE-743 and MAPS trials (clinical trial cohorts).

**Results:**

244 patients were enrolled between 7 April 2017 and 1 March 2022. The cohort was predominantly male (195 out of 244; 80%) with a median age of 74 years. Pleural disease and epithelioid subtypes were most prevalent. ASSESS-meso participants were more similar to the real-world population with regard to age, performance status, disease site and stage than the clinical trial population. ASSESS-meso participants were more likely to be formally staged and less likely to have undifferentiated histology compared with the real-world cohort, possibly reflecting high rates of discussion of ASSESS-meso participants at regional mesothelioma multidisciplinary team meetings. As expected, poorer performance status, non-epithelioid histology and neutrophil–lymphocyte ratio were associated with shorter survival in the adjusted analysis.

**Conclusion:**

ASSESS-meso is representative of the UK mesothelioma population. Future outputs from the cohort will help characterise different mesothelioma phenotypes with high external validity.

## Tweetable abstract

**Interim report from prospective UK ASSESS-meso cohort demonstrates recruitment of a representative population of mesothelioma patients. Great research inclusivity, and assurance that future outputs from this study will inform real-world patient care.** https://bit.ly/3FI1dGk

## Introduction

Mesothelioma is an aggressive malignancy of the serosal surface, most commonly affecting the pleura (pleural mesothelioma (PM)). It is usually incurable, with median survival of 8 to 12 months from diagnosis, and only marginal survival benefits offered by chemotherapy [1–3]. Significant research effort over the past decade has resulted in recent expansion of effective treatment options to include frontline combination immunotherapy, single-agent second-line immunotherapy and additional vascular endothelial growth factor antagonists alongside chemotherapy [4–6].

**Lessons for clinicians**

• First report from the prospective, longitudinal, UK mesothelioma cohort, ASSESS-meso

• Participant characteristics were similar to those of real-world populations

• Survival was associated with performance status, histology, neutrophil–lymphocyte ratio and Brims score

• Future outputs from ASSES-meso are likely to have high external validity

Despite these valuable advances, it is recognised that mesothelioma responds variably to systemic anticancer therapy (SACT) [7], due to heterogeneity in underlying histopathological subtype [8–16] and molecular profile [17–23]. Even without SACT, there is significant variation in disease course and overall survival of patients with PM, with several factors associated with progression and prognosis, including patients’ age and performance status, symptoms, tumour stage and markers of systemic inflammation [11–16, 24–32]. Unfortunately, due to strict (albeit necessary) inclusion criteria, clinical trial populations often do not reflect the full heterogeneity of PM, which can limit the generalisability of results. For example, 50% of PM patients in an Australian study did not meet the eligibility criteria for a recent phase III treatment trial [33]. This lack of external validity also explains the discrepancy between treatment response rates seen in early chemotherapy trials and real-world delivery programmes [3, 34].

Prospective data from large representative patient cohorts are needed to fully elucidate and describe the varying PM disease courses and clinical phenotypes, and to provide a resource for pragmatic, generalisable clinical trials. National audit data (including the UK National Mesothelioma Audit) and disease registries provide population-level data and aggregated outcome information, but prospective, longitudinal monitoring of individual patients provides more detailed data to inform specific phenotyping. Discovery and validation of monitoring biomarkers for response evaluation could facilitate a paradigm shift to personalised treatment, similar to the approach for nonsmall cell lung cancer [35].

ASSESS-meso was designed to address this research gap [36]. In this paper, we report the pre-specified interim analysis of ASSESS-meso, undertaken when recruitment reached 25% of target. We aimed to describe the baseline characteristics of ASSESS-meso participants and to evaluate external validity by comparing the cohort with clinical trial populations (participants in recent phase III clinical trials) and real-world cohorts (the UK National Mesothelioma Audit).

## Material and methods

### Study design and participants

ASSESS-meso is an ongoing, multicentre, prospective, longitudinal, observational cohort study of people with mesothelioma, the protocol of which has been published elsewhere [36]. Eligible participants were recruited at 14 hospitals across the UK (12 in England, one in Scotland, one in Wales), including a mix of secondary and tertiary care providers. Eligible participants were adults with a multidisciplinary team (MDT)-ratified diagnosis of mesothelioma (any disease site, histological, cytological or clinico-radiological diagnoses) who were able to provide consent and willing to attend regular follow-up. Recruitment commenced on 4 July 2017 and is ongoing. Data for this interim analysis was extracted on 1 March 2022. All participants provided informed consent and enrolled within 12 weeks of receiving a diagnosis of mesothelioma.

### Data collection and follow-up

Participants were enrolled at clinic appointments, including respiratory, pleural, oncology and cancer specialist nurse-led clinics. Follow-up visits were scheduled flexibly to align with routine clinical follow-up, with data collection occurring 3-monthly as a minimum. Study visits occurred face-to-face wherever possible, with the option for virtual or telephone follow-up if face-to-face appointments were not possible (*e.g.* due to declining patient mobility or COVID-19 restrictions). Data were collected by research nurses onto the online study database (REDCap, Vanderbilt University, USA). Study follow-up continued from enrolment to death or withdrawal from the study.

### Variables and measurements

The primary outcome of the study was survival, measured from date of diagnosis with mesothelioma (defined as date diagnosis confirmed at MDT) to date of death (or censored on the date of data lock). The main study analysis, to be undertaken at study completion (1 year after recruitment of the final participant), will explore factors associated with survival, including baseline participant and tumour characteristics, symptom scores at baseline and longitudinally, biological parameters including blood and pleural fluid inflammatory markers, genomics, proteomics and metabolomics, radiological markers of disease severity and progression, and oncological and specialist treatments received, with adjustment for known confounder and mediator variables. A complete list of variables, measurements and handling methods are provided in the supplementary material.

This paper reports the results of a pre-specified interim analysis evaluating the external validity of the cohort, undertaken when recruitment reached 25% of target. Baseline demographic and clinical data were reported for all participants, and compared with matched variables from two clinical trial populations and a real-world cohort.

### Comparator populations

Baseline ASSESS-meso data were compared with participant characteristics from two recent phase III randomised controlled trials (MAPS [6] and CHECKMATE-743 [4]), chosen on the basis that their frontline setting matched enrolment timelines in ASSESS-meso. Data were extracted from published manuscripts, online appendices and supplementary material.

Real-world comparator data were obtained from the 2020 UK National Mesothelioma Audit Report (which included data from all cases of mesothelioma seen in UK hospitals between 2016 and 2018). Data were extracted from the raw dataset for the audit, which is available online [37].

### Statistical analysis

Descriptive statistics were used to report the baseline characteristics of the ASSESS-meso cohort. Categorical data were presented as numbers (with percentages) and continuous data as means (95% confidence intervals (CI)) or medians (with range and/or interquartile range (IQR)), according to distribution. Missing data were reported for each variable, but as no inferential analyses were undertaken and no assumptions were made about patterns of missing data, statistical methods to address incompleteness were not applied.

Survival was reported for the whole cohort. Cox proportional hazards model was used to evaluate the relationship between survival and known prognostic variables, including age, sex, performance status, disease site, tumour histology and stage and neutrophil–lymphocyte ratio (NLR), as crude analyses and then adjusted for aforementioned variables. The relationship between Brims score [38] and survival was assessed in a separate Cox model, crude and adjusted for age, sex, disease site, tumour stage and NLR. Variables used to calculate Brims score were excluded from the adjusted model to avoid collinearity.

Patient characteristics, disease variables and survival were tabulated and compared between ASSESS-meso and the comparator populations. STATA (version 17.0; Stata Corp. LP, College Station, TX, USA) was used for analyses.

### Ethical considerations and approval

This study was approved by the Research Ethics Committee South West – Central Bristol (17-SW-0019) and Health Research Authority (IRAS ID 220360). All participants provided informed consent for participation.

## Results

### Participant demographics

Between 4 July 2017 and 1 March 2022, 244 patients enrolled in the study. All participants enrolled within 12 weeks of receiving a diagnosis of mesothelioma (median time from MDT diagnosis to enrolment 30.5 days (IQR 18–56)). All patients were discussed at the MDT prior to enrolment. For 210 patients, enrolled at 11of 14 sites, discussion occurred at a regional mesothelioma MDT, whilst the remaining 34 patients were discussed at local lung cancer MDTs. Time to enrolment was similar for patients regardless of MDT.

Participant demographics and baseline characteristics are shown in table 1. The majority of participants were male (195, 79.9%) and over the age of 65 (219, 89.8%). Most patients had an Eastern Cooperative Oncology Group (ECOG) performance status of 0–1 (161 of 194; 82.9%), despite 192 comorbidities recorded across the cohort, and 98 of 244 (40.2%) having two or more comorbidities. Asbestos exposure was recalled in 205 (84.0%), with most people describing direct exposure (112, 45.9%). Many women, (18 of 49; 36.7%) recalled no asbestos exposure. Of those that did (31 of 49; 63.3%), very few had active exposure through working directly with and in close proximity to asbestos (five of 31; 16.1%). Instead, those exposed largely reported environmental exposure *via* the presence of (undisturbed) asbestos in the walls/ceilings of their home or workplace (nine of 31; 29.0%) or passive exposure through spending time in an enclosed space where other people were working with asbestos, generating air-borne dust and fibres (17 of 31; 54.8%).

**TABLE 1:** Participant characteristics

**Age years, median (range)**	74.0 (53–88)
**Age by category**	
<65 years	25 (10.3)
65–69 years	34 (13.9)
70–74 years	70 (28.7)
75–79 years	56 (23.0)
80–84 years	39 (16.0)
≥85 years	20 (8.2)
**Sex**	
Male	195 (79.9)
Female	49 (20.1)
**Body mass index kg·m^−2^, median (IQR)**	25.3 (23.4–28.2)
**Performance status**	
0	53 (21.7)
1	108 (44.3)
2	28 (11.5)
3	5 (2.0)
4	0 (0)
Unknown/missing	50 (20.5)
**Smoking**	
Smoker (ex/current)	77 (31.6)
Never	53 (21.7)
Unknown/missing	114 (46.7)
**Asbestos exposure**	
Direct exposure (*i.e.* participant was working directly with asbestos, generating dust or fibres)	112 (45.9)
Indirect exposure (*i.e.* participant worked in an environment where asbestos was used and dust/fibres were in the air, but participant was not working with it directly)	57 (23.4)
Environmental (*i.e.* asbestos present in walls/ceilings of home or workplace, but was not knowingly disturbed)	36 (14.8)
No exposure recalled	37 (15.2)
Unknown/missing	2 (0.8)
**Past medical history**	
Cancer elsewhere	54 (22.5)
Previous	40
Current	14
COPD	37 (15.2)
Diabetes	29 (11.9)
Ischaemic heart disease	28 (11.6)
Atrial fibrillation	22 (9.1)
Chronic kidney disease	12 (5.0)
Previous lung infection	10 (4.2)
**Number of comorbidities^#^**	
0	60 (24.6)
1	86 (35.2)
2–4	87 (35.6)
≥5	11 (4.5)
**Symptoms at diagnosis**	
Breathlessness	200 (82.0)
Cough	92 (37.7)
Chest pain	86 (35.2)
Weight loss	73 (29.9)
Lethargy	51 (20.8)
Sweats	31 (12.7)
Anorexia	26 (10.6)

Data are presented as n (%) unless otherwise specified. ^#^: full list of comorbidities provided in supplementary material.

### Disease characteristics

Disease-specific details are shown in table 2. The most common disease site was the pleura (233, 95.5%). Histological diagnosis was obtained in 209 (85.7%) participants, with diagnosis based on cytology in 19 (7.8%), and in 14 (5.7%) an MDT consensus clinic-radiological diagnosis was made (data were missing for two participants; 0.8%). Of 209 participants who underwent biopsy, 95 (45.4%) had a local anaesthetic thoracoscopy, 35 (16.7%) had video-assisted thoracic surgical biopsy, 34 (16.2%) ultrasound-guided biopsy, 27 (12.9%) CT-guided biopsy and 14 (6.7%) *via* other methods, including laparoscopy and mediastinoscopy. Data on biopsy method were missing in four out of 209 (1.9%).

**TABLE 2:** Disease characteristics

**Site**	
Pleural	233 (95.5)
Peritoneal	6 (2.5)
Pericardial	4 (1.6)
Other^#^	1 (0.4)
**Histology**	
Epithelioid	163 (66.8)
Sarcomatoid/desmoplastic	25 (10.2)
Biphasic	16 (6.6)
Mesothelioma NOS	5 (2.0)
Cytological diagnosis	19 (7.8)
Biopsy/cytology not obtained	14 (5.7)
Unknown/ missing	2 (0.8)
**TNM stage (v8)**	
IA	53 (21.7)
IB	34 (13.9)
II	17 (7.0)
IIIA	23 (9.4)
IIIB	43 (17.6)
IV	21 (8.6)
Non-pleural disease	11 (4.5)
Unknown/missing^¶^	42 (17.2)
**NLR, median (IQR)**	4.2 (2.9–6.7)
<4	100 (41.0)
≥4	119 (48.8)
Not calculable	25 (10.2)

Data are presented as n (%) unless otherwise specified. NOS: not otherwise specified; TNM: tumour, node, metastasis; NLR: neutrophil-to-lymphocyte ratio. ^#^: mediastinal mass, unclear whether pleural or pericardial; ^¶^: including no visible disease on computed tomography.

Epithelioid mesothelioma was the most frequently reported histological subtype (163 of 244; 66.8%,), followed by sarcomatoid or desmoplastic (25, 10.2%) and biphasic disease (16, 6.6%). The majority of tumours were stage 1 (87, 35.6%) or stage 3 (66, 27.0%), but stage was not recorded in 42 (17.2%), despite most cases (35 of 42; 83.3%) having been discussed at a regional mesothelioma MDT.

### Symptoms

The most common presenting symptom was breathlessness, which was reported by 200 participants (82.0%). Cough (92, 37.7%), chest pain (86, 35.2%) and weight loss (73, 29.9%) were also frequently reported. Lethargy (51, 20.8%), sweats (31, 12.7%) and anorexia (26, 10.6%) were reported by fewer people, but contributed to the overall symptom burden nonetheless. Symptoms had been present for at least 1 month in 107 people (43.9%) and over 3 months in 87 (35.6%) (data missing for five people; 2%). Time from symptom onset to diagnosis was not associated with geographical site, nor availability of specialist mesothelioma MDT.

The severity of specific symptoms was graded using a 100 mm visual analogue scale (VAS), where a score of 0 reflects no symptom and a score of 100 represents the worst severity imaginable. In general, participant-reported scores for breathlessness, chest pain and sweats were low at baseline. Median VAS score for breathlessness was 9.25 mm (IQR 1.88–32.5), chest pain 3 mm (IQR 0–13) and sweats 1.25 mm (IQR 0–10.5). However, symptom score distributions were positively skewed, with 14 of 244 (5.7%) participants reporting symptom scores ≥80 across all three symptoms.

### Treatment

In total, 122 (50.0%) participants received between one and six cycles of chemotherapy (median number of cycles three). Most participants received carboplatin and pemetrexed doublet (95 of 244, 38.9%), many fewer received cisplatin with pemetrexed combination (22, 9.0%). The remaining participants received single agent pemetrexed (two, 0.8%), pemetrexed, carboplatin and bevacizumab (one, 0.4%), and pemetrexed, carboplatin, bevacizumab and atezolizumab (one, 0.4%) One participant received ramucirumab (0.4%) and one received an unspecified “trial drug” (0.4%). 13 participants underwent surgery, all in the form of extended pleurectomy/decortication within the MARS2 trial [39]. Treatment regimen was not recorded for one participant.

Immunotherapy was given to 57 (23.4%) participants, usually nivolumab with ipilimumab (23; 9.4%) or nivolumab alone (26; 10.7%). One participant apiece received pembrolizumab (0.4%) and atezolizumab (0.4%), whilst immunotherapy agents were delivered but not named for six of 244 (2.5%). Of the participants who received immunotherapy, 32 of 57 (56.1%) received prior first-line chemotherapy, whilst 25 of 57 (43.9%) received immunotherapy upfront, 12 of whom received chemotherapy subsequently, after disease progression.

46 people (18.9%) declined an offer of treatment, including 31 (12.7%) who chose not to receive chemotherapy, four (1.6%) who declined surgery (both within and without the MARS2 trial [39]), seven (2.9%) who turned down immunotherapy, two (0.8%) who did not want to enrol in the MIST trial [40] and two (0.8%) who declined all forms of treatment. Reasons for declining treatment or trial participation included prioritising quality of life (eight, 3.3%), opting for watchful waiting during an asymptomatic period (10, 4.1%), concern around potential side-effects (12, 4.9%), anxiety/claustrophobia (three, 1.2%) and other priorities (three, 1.2%) such as carer responsibilities, plans to do a sky jump and plans for a round-the-world trip.

### Survival

Survival was censored on 1 March 22, at which point 107 of 224 (43.7%) patients had died. Median survival was 313.5 days (IQR 162–488), with living participants followed up for a median of 308 days (range 29–1489). Factors associated with shorter survival in the unadjusted analysis included age (crude HR 1.03, 95% CI 1.01–1.06, p=0.02), performance status of ≥2 (crude HR 3.02, 95% CI 1.90–4.80, p<0.001), non-epithelioid histology (crude HR 1.71, 95% CI 1.06–2.75, 0=0.03), stage (crude HR 1.36, 95% CI 1.11–1.66, p=0.003), absolute NLR (crude HR 1.07, 95% CI 1.04–1.11, p<0.001) and NLR >4 (crude HR 1.69, 95% CI 1.13–2.52, p=0.011). In the adjusted model, performance status ≥2 (adjusted HR 3.96, 95% CI 2.14–7.30, p<0.001), non-epithelioid histology (adjusted HR 1.83, 95% CI 1.01–3.31, p=0.045) and absolute NLR (adjusted HR 1.07, 95% CI 1.03–1.11, p=0.001) were associated with shorter survival. Neither sex nor disease site were associated with a survival difference, nor was discussion at regional mesothelioma MDTs (see online supplementary material). A small subgroup of participants (n=22, 9.0%) with performance status of 0, epithelioid tumours and NLR <4 had longer median survival at 16.3 months (IQR 10.1–25.3), confirming clinical observations that certain patients with mesothelioma have better prognoses and warrant further investigation.

Brims prognostic score could be calculated for 192 participants. Higher Brims score was associated with shorter survival, although the survival gradient between groups was less pronounced than in the original dataset (table 3). Mortality risk increased with higher Brims group (unadjusted HR 2.11, 95% CI 1.6–42.71, p<0.001; adjusted HR 2.05, 95% CI 1.55–2.72, p<0.001).

**TABLE 3:** Overall survival by Brims score, compared with predicted survival per group from original Brims cohort [38]

**Brims score**	**n (%)**	**Median survival, months (IQR)**	**Predicted survival based on Brims score, months (IQR) [38]**
**1**	4 (1.6)	19.4 (10.8–26.6)	34.0 (22.9 –47.0)
**2**	77 (31.4)	12.2 (6.6–23.4)	17.7 (11.6–25.9)
**3**	33 (13.5)	11.9 (5.6–17.0)	12.0 (6.0–20.6)
**4**	78 (31.8)	9.7 (5.0–12.9)	7.4 (3.3–11.1)
**Unable to calculate**	53 (21.6)	78.0 (4.3–12.6)	

IQR: interquartile range.

### Comparison with other populations

ASSESS-meso participants were similar to the population of people with mesothelioma as recorded in the UK National Mesothelioma Audit in terms of age, sex and disease site (table 4). In contrast, clinical trial populations tended to be younger, with a higher proportion of female participants, and with a focus purely on pleural mesothelioma. Participants in ASSES-meso had a wider range of performance statuses than those in clinical trials, although ASSESS-meso included fewer people with performance status 3–4 than the national audit, potentially reflecting a lower likelihood of hospital-based follow-up in people with higher performance status. Interestingly, the majority of CHECKMATE-743 participants had advanced stage disease, whilst ASSESS-meso had a predominance of stage 1 and 3 tumours. Missing stage and smoking data in the audit, and ASSESS-meso to a lesser degree, precluded further meaningful comparison. Unlike clinical trial populations, ASSESS-meso included a large group of people who did not receive SACT, although treatment rates were a little higher than reported in the UK Mesothelioma Audit.

**TABLE 4:** Characteristics of ASSESS-meso cohort compared with patients from the National Mesothelioma Audit 2020 report, and two recent first-line setting clinical trial populations

	**ASSESS-meso**	**National Mesothelioma** **Audit [37], 2020**	**MAPS [6], 2016**	**CHECKMATE 743 [4], 2021**
**Number of participants**	244	6950^#^	448	605
**Age years, median (IQR)**	74.0 (70.0–79.0)	76.0 (70.0–82.0)	65.7 (61.3–70.2)	69.0 (64.0–75.0)
**Sex**				
Male	195 (79.9)	83.3	338 (75.4)	467 (77.2)
Female	49 (20.1)	16.7	110 (24.6)	138 (22.8)
**Performance status**				
0	53 (21.7)	15.2	433 (96.7)^¶^	242 (40.0)
1	108 (44.3)	36.2		362 (60.0)
2	28 (11.5)	15.7	15 (3.3)	0
3	5 (2.0)	11.3	0	0
4	0	2.2	0	0
Missing/unknown	50 (20.5)	19.4	0	0
**Smoking**				
Smoker (ex/current)	77 (31.6)	-	254 (56.7)	344 (56.9)
Never	53 (21.7)	-	194 (43.3)	261 (41.2)
Unknown/missing	114 (46.7)	-	0	2.0
**Site**				
Pleural	233 (95.1)	96.4^+^	448 (100)	605 (100)
Non-pleural	11 (4.9)	3.6^+^	0	0
Missing	0	0^+^	0	0
**Histology**				
Epithelioid (including cytological diagnoses)	182 (74.6)	42.5	361 (80.6)	456 (75.4)
Sarcomatoid/desmoplastic	25 (10.2)	9.9	87 (19.4)^§^	71 (11.7)
Biphasic	16 (6.6)	-		78 (12.9)
Mesothelioma NOS	5 (2.0)	46.9	0	0
Unknown/missing	16 (6.5)	0.7	0	0
**Stage**				
1	87 (35.6)	17.4	-	32 (5.3)
2	17 (7.0)	6.6	-	45 (7.4)
3	66 (27.0)	20.9	-	209 (34.5)
4	21 (8.6)	20.6	-	309 (51.1)
Non-pleural disease	11 (4.5)		-	-
Unknown/not staged	42 (17.2)	34.6	-	10 (1.7)
**Treatment**				
Received chemo- or immunotherapy	135 (55.1)	2752 (39.6)	448 (100)	605 (100)

Data are presented as n (%) unless otherwise specified. IQR: interquartile range; NOS: not otherwise specified. ^#^: demographics reported for pleural mesothelioma; ^¶^: performance status 0–1 reported together; ^+^: total n=7210, including peritoneal mesothelioma; ^§^: sarcomatoid and biphasic reported together.

## Discussion

This is a pre-specified interim analysis of the first 244 participants enrolled in the ASSESS-meso cohort study. These initial data showed that the cohort population is reflective of the real-world patient population, with close similarities to the 2020 UK National Mesothelioma Audit [37]. Compared with the populations of two recent frontline clinical trials, the ASSESS-meso cohort included more older people, more people of higher performance status and more people with non-pleural disease. Additionally, by including people who are not receiving or have declined SACT, ASSESS-meso offers unique and important insight into the disease course and participant experience of this understudied group.

The differences highlighted here between clinical trial populations and real-world patient groups reflect the perennial tension between efficacy trials of new pharmaceutical agents and pragmatic implementation/effectiveness studies. The inclusion of MAPS and CHECKMATE-743 as comparators in this study was not intended negatively; indeed, both trials were robustly designed and delivered, and between them advanced the treatment landscape for mesothelioma dramatically after a decade of negative results. It is recognised that the safety requirements of clinical trials of investigational products demand more stringent eligibility criteria, creating an unavoidable selection bias. The unavoidable consequence of this, however, is that positive clinical trial outcomes are not always replicated once drug availability expands to include more diverse populations. For example, in the original EMPHACIS trial of cisplatin and pemetrexed, overall response rates were 41% but dropped to 26% when the regimen was made available to clinical populations *via* the International Expanded Access Programme [3, 34]. The availability of representative patient cohorts such as ASSESS-meso facilitates the use of pragmatic methodologies, such as Trials within Cohorts (TwiCs) [41–43], for future clinical trials, potentially reducing this issue.

The finding that initial recruitment to ASSESS-meso was representative of the wider UK mesothelioma population is encouraging. Study recruitment and data collection is scheduled to continue for another 5 years, with several substudies underway within the main cohort. These include collaborations across the Cancer Research UK funded PREDICT-meso network (https://www.predictmeso.com/our-research-teams/), using biological samples collected within ASSESS-meso to explore genomic, transcriptomic and molecular expression and evolution from pre-malignant states to mesothelioma; diagnostic and response biomarkers such as circulating tumour DNA and serum mesothelin; and the role and importance of the pleural microbiome. This ongoing work promises to provide valuable insight into the biology of mesothelioma development and progression, and may yield new tests to inform clinical practice, *e.g.* by identifying patients with rapidly progressive disease, or those who may respond better to certain treatment modalities. Confirmation that the samples under investigation have been collected from a representative patient group ensures these future outputs will have high external validity and clinical utility.

### Strengths and limitations

As demonstrated, ASSESS-meso has enrolled a representative sample of the mesothelioma population. As well as descriptive data on participants’ characteristics, summary statistics of our cohort were consistent with existing, established understanding of the disease. Specifically, the factors associated with shorter survival, namely non-epithelioid histology, poor performance status and inflammatory markers in the form of raised NLR, were consistent with prior evidence [2, 44, 45]. The ability of Brims score, a prognostic model derived from two international clinical cohorts, to predict survival within ASSESS-meso adds further validation to the scoring system, and further supports the external validity of ASSESS-meso. The reduced survival gradient across Brims score groups in ASSESS-meso may be a result of the smaller participant numbers within Group 1 or may reflect the immaturity of the cohort, in that more than half of participants remained alive at the time of analysis. Similarly, the lack of a significant relationship demonstrated in multivariable analysis for certain known prognostic factors, *e.g.* age and stage, was likely a result of this being an interim analysis and not formally powered for a fully adjusted survival model. The study is scheduled to continue until 700 participants have been recruited and followed up for a minimum of 12 months, hence this issue will not affect the final analysis.

Another strength related to the high rates of histological subtyping, with low numbers of “Mesothelioma, NOS” (not otherwise specified). This is likely due to the majority of participants being discussed at regional mesothelioma MDTs, allowing review of histological samples and access to additional diagnostic and immunohistochemical tests. The use of cytology to confirm a diagnosis (usually alongside a consistent clinical presentation and supportive radiology) is another benefit offered by regional mesothelioma MDTs.

Somewhat disappointingly, discussion at regional mesothelioma MDT did not guarantee reliable recording of staging data, with 17% of participants entering the study without a recorded stage. However, the prevalence of unstaged disease was lower in our cohort than in the real-world dataset where 34% of patient did not have a formal stage recorded. This is likely a result of recognised limitations with current staging systems that were derived from pathological assessment of tumour invasion, and can be challenging to assess on radiological imaging. We hypothesise that the high proportion of stage 1 tumours in our cohort and the clustering of staging around stages 1 and 3 reflects these challenges, as well as potentially resulting from inter-report variability. Acquisition of baseline images for central review by an independent radiologist is planned, and will improve completion rates and provide consistent staging data for future analyses.

Missing data is a common problem in longitudinal cohorts and was in evidence here, specifically with regard to performance status and smoking status. The COVID-19 pandemic had some influence, as data collection was challenging during this period due to staff redeployment, reduced face-to-face clinical and research activity, and (appropriate) national prioritisation of COVID studies. The overall impact of missing data will be evaluated in the full dataset prior to final analysis, but it is likely that statistical methods will be required to address this. Helpfully, noting this finding in the current interim analysis has provided a stimulus to increase data quality and to reduce missing data for future participants.

COVID-19 also impacted recruitment to ASSESS-meso, for many of the reasons documented above. As well as limiting enrolment activities, the pandemic prevented additional sites opening, further slowing recruitment. Fortunately, research activity has accelerated post-pandemic, with seven new sites opening since January 2022, and recruitment exceeding 60% of target as of June 2023.

### Conclusions

ASSESS-meso has achieved its initial aim to enrol a representative cohort of mesothelioma patients. Future outputs from this study will have high external validity for the UK mesothelioma population.

## Supplementary material

10.1183/23120541.00467-2023.Supp1 **Please note:** supplementary material is not edited by the Editorial Office, and is uploaded as it has been supplied by the author.
Supplementary material 00467-2023.SUPPLEMENT
