# Supplementary material for: Reflecting real-world patients with mesothelioma in research: an interim report of baseline characteristics from the ASSESS-meso cohort
Source: ERJ Open Res. 2023 Dec 27;9(6):00467-2023. doi: 10.1183/23120541.00467-2023 (PMC10763666; doi:10.1183/23120541.00467-2023)
Supplement: Supplementary file 1 [file 00467-2023.SUPPLEMENT.pdf]

Supplementary Appendix

Contents

- STROBE checklist
- Eligibility criteria
- Data collection and handling
- Survival analysis

STROBE CHECKLIST

STROBE Statement—Checklist of items that should be included in reports of ***cohort studies***

	Item No	Recommendation
**Title and abstract**	1	(*a*) Indicate the study's design with a commonly used term in the title or the abstract<br><br>Included in page 1, lines 1-2.
		(*b*) Provide in the abstract an informative and balanced summary of what was done and what was found<br><br>Included in pages 1-2, lines 15-43.
**Introduction**		
Background/rationale	2	Explain the scientific background and rationale for the investigation being reported<br><br>Included in pages 3-4, lines 59-91.
Objectives	3	State specific objectives, including any prespecified hypotheses<br><br>Included in page 4, lines 87-91.
**Methods**		
Study design	4	Present key elements of study design early in the paper<br><br>Included in page 4, lines 95-103.<br><br>Also available in the study protocol (Conway et al, 2022).
Setting	5	Describe the setting, locations, and relevant dates, including periods of recruitment, exposure, follow-up, and data collection<br><br>Included in page 4, lines 95-110
Participants	6	(*a*) Give the eligibility criteria, and the sources and methods of selection of participants. Describe methods of follow-up<br><br>Included in page 4, lines 95-110.
		(*b*) For matched studies, give matching criteria and number of exposed and unexposed<br><br>n/a
Variables	7	Clearly define all outcomes, exposures, predictors, potential confounders, and effect modifiers. Give diagnostic criteria, if applicable<br><br>Included in page 5, lines 115-138.
Data sources/ measurement	8*	For each variable of interest, give sources of data and details of methods of assessment (measurement). Describe comparability of assessment methods if there is more than one group<br><br>Included in page 5, lines 115-138.

Bias	9	Describe any efforts to address potential sources of bias<br><br>Included in page 5, lines 130-138
Study size	10	Explain how the study size was arrived at<br><br>Outlined in the study protocol (Conway et al, 2022)
Quantitative variables	11	Explain how quantitative variables were handled in the analyses. If applicable, describe which groupings were chosen and why<br><br>Included in page 5, lines 114-128 and more extensively outlined in the study protocol (Conway et al, 2022)
Statistical methods	12	(*a*) Describe all statistical methods, including those used to control for confounding<br><br>Included in pages 5-6, lines 140-158
		(*b*) Describe any methods used to examine subgroups and interactions<br><br>Subgroup interactions not examined
		(*c*) Explain how missing data were addressed<br><br>The proportion of missing data for each variable were included.
		(*d*) If applicable, explain how loss to follow-up was addressed<br><br>N/a; no patients withdrew from the study.
		(*e*) Describe any sensitivity analyses<br><br>n/a
**Results**		
Participants	13*	(a) Report numbers of individuals at each stage of study—eg numbers potentially eligible, examined for eligibility, confirmed eligible, included in the study, completing follow-up, and analysed<br><br>Analysed population described on page 7 lines 167-183.<br><br>Follow-up: n/a - this study examines primarily demographics and baseline characteristics but limited survival data is reported (with censoring on 1st March 2022) on pages 9-10.
		(b) Give reasons for non-participation at each stage<br><br>As participants were recruited from standard clinical care. Reasons for why patients who did not consent to enrolment chose not to participate were not recorded to sufficient level to allow meaningful conclusions to be drawn.
		(c) Consider use of a flow diagram<br><br>Not required for this particular study.
Descriptive data	14*	(a) Give characteristics of study participants (eg demographic, clinical, social) and information on exposures and potential confounders<br><br>Included throughout pages 7, as well as Tables 1 and 2.

		(b) Indicate number of participants with missing data for each variable of interest<br><br>Included throughout pages 7-10, as well as Tables 1-4 (which have dedicated lines for each missing variable).
		(c) Summarise follow-up time (eg, average and total amount)<br><br>Included on pages 4-5 (Methods), and the the survival sub-section of Results, pages 9-10.
Outcome data	15*	Report numbers of outcome events or summary measures over time<br><br>Listed in Survival section, pages 9-10, lines 241-259.
Main results	16	(*a*) Give unadjusted estimates and, if applicable, confounder-adjusted estimates and their precision (eg, 95% confidence interval). Make clear which confounders were adjusted for and why they were included<br><br>Both unadjusted and adjusted HR for survival are listed in results on pages 9-10, lines 243-254.<br><br>Reasons for why particular confounders were adjusted for are listed in the statistical analysis section of the methods on page 6.
		(*b*) Report category boundaries when continuous variables were categorized<br><br>Not applicable for this particular study.
		(*c*) If relevant, consider translating estimates of relative risk into absolute risk for a meaningful time period<br><br>Not applicable for this particular study.
Other analyses	17	Report other analyses done—eg analyses of subgroups and interactions, and sensitivity analyses<br><br>Not applicable for this particular study.
**Discussion**		
Key results	18	Summarise key results with reference to study objectives<br><br>Discussed on pages 10-11, line 276-283.
Limitations	19	Discuss limitations of the study, taking into account sources of potential bias or imprecision. Discuss both direction and magnitude of any potential bias<br><br>Limitations and missing data and address in lines 312-350 on pages 12-13.
Interpretation	20	Give a cautious overall interpretation of results considering objectives, limitations, multiplicity of analyses, results from similar studies, and other relevant evidence<br><br>Discussed on page 13, lines 353-354.
Generalisability	21	Discuss the generalisability (external validity) of the study results<br><br>Discussed on page 13, lines 353-354.

**Other information**		
Funding	22	Give the source of funding and the role of the funders for the present study and, if applicable, for the original study on which the present article is based<br><br>Discussed in the Funding section, lines 367-368 of page 13.

*Give information separately for exposed and unexposed groups.

Note: An Explanation and Elaboration article discusses each checklist item and gives methodological background and published examples of transparent reporting. The STROBE checklist is best used in conjunction with this article (freely available on the Web sites of PLoS Medicine at http://www.plosmedicine.org/, Annals of Internal Medicine at http://www.annals.org/, and Epidemiology at http://www.epidem.com/). Information on the STROBE Initiative is available at http://www.strobe-statement.org.

ELIGIBILITY CRITERIA

Inclusion criteria

Participants must meet *all* of the following criteria:

- histological, cytological, clinico-pathological or radiological diagnosis of mesothelioma, confirmed by local lung cancer or regional mesothelioma multidisciplinary team (MDT).
- willing and able to comply with study follow-up assessments.
- willing & able to provide written informed consent.

Exclusion criteria

Participants must meet *none* of the following criteria:

- age <18 years old.
- unable to give written informed consent.
- declines ongoing hospital follow-up.

DATA COLLECTION AND HANDLING

Demographics

- Date of enrolment into study
- Age (continuous; years)
- Sex (binary; male/female)
- Weight (continuous; kilograms)
- Height (continuous; metres)
- BMI (calculated as weight/ height^2; kg/m^2)
- Weight loss since enrolment (total amount and % of baseline weight, continuous; kilograms)
- Smoking status (categorical; current smoker, ex-smoker, never smoker (less than 1 tobacco product a day for less than 1 year))
- Alcohol consumption, (continuous; number of days alcohol consumed per week and number of units consumed per week)
- Asbestos exposure (categorical; None recalled, environmental exposure (asbestos in walls/ceilings of home or workplace, but not disturbed), passive exposure (worked in an environment where others were working with asbestos, dust/fibres in the air but participant was not working with it directly), active exposure (participant worked directly with asbestos, generating dust or fibres).
- Post code (descriptive, used to calculate Index of Multiple Deprivation)
- Co-habitation (binary; yes/no)
- Performance status (ordinal, ECOG 0-4). Handled as binary (PS<2 or ≥2) for some analyses.
- Co-morbidities (binary; yes/no for 20 common conditions, with descriptive text field for capture of non-listed conditions. The 20 listed conditions are asthma, Chronic Obstructive Pulmonary Disease (COPD), interstitial lung disease, bronchiectasis, pulmonary hypertension, pulmonary emboli/ deep vein thrombosis (PE/DVT), pleural infection, ischaemic heart disease, Atrial Fibrillation (AF), congestive cardiac failure, valvular heart disease, peripheral vascular disease, diabetes mellitus, chronic kidney disease, current or previous cancer elsewhere, and gastroenterological, neurological, endocrine, cardiac or respiratory disease not otherwise listed

Disease characteristics

- Date of diagnosis
- Date of MDT confirmation of diagnosis
- Site of disease (categorical; pleural, peritoneal, pericardial, other)
- Side of disease (categorical; left, right, bilateral)
- Histological sub-type (categorical; epithelioid, sarcomatoid, biphasic, desmoplastic, unspecified, other). Handled as binary (epithelioid/ non-epithelioid) for some analyses.
- Disease stage (categorical; TNM stage 8)
- Diagnostic method (categorical; histological, cytological, clinic-radiological (based on MDT consensus))
- Biopsy method, if obtained (categorical; ultrasound-guided biopsy, CT-guided biopsy, local anaesthetic thoracoscopy, video-assisted thoracoscopy/ surgical biopsy, other).

Baseline symptoms

- Symptoms (binary; yes/no for chest pain, breathlessness, cough, sweats, lethargy, anorexia, weight loss)
- Duration of symptoms (categorical; <1 month, 1-3 months, >3 months)

- Symptom severity for breathless, chest pain and sweats (continuous; 10cm visual analogue scales)

Medical interventions and treatments

- Presence of indwelling pleural catheter (IPC) (binary; yes/no)
    - Laterality of IPC (categorical; right/ left/ bilateral)
    - Date of insertion
    - Date of removal
- Previous pleural aspiration (binary; yes/no)
    - Diagnostic vs therapeutic (binary)
    - Date of aspiration
    - Laterality (categorical; right/ left/ bilateral)
    - Volume of fluid aspirated (continuous; millimetres)
    - Number of pleural aspirations (continuous)
- Previous intercostal chest drain (binary; yes/no)
    - Date of chest drain
    - Laterality (categorical; right/ left/ bilateral)
    - Total volume of fluid drained (continuous; millimetres)
    - Number of chest drains (continuous)
- Previous pleurodesis (binary; yes/no)
    - Date of pleurodesis
    - Laterality (categorical; right/ left/ bilateral)
    - Delivery method (categorical; talc slurry, thoracoscopic poudrage; surgical pleurodesis)
    - Pleurodesis success, defined as resolution of pleural fluid on radiological imaging and/or no further pleural interventions required (binary; yes/no)
- Previous surgical intervention (binary; yes/no)
    - Type of surgery (free text)
    - Date of surgery
    - Laterality (categorical; right/ left/ bilateral)
- Chemotherapy history (binary; yes/no), with details of regimen and dates
- Radiotherapy (binary; yes/no), with details of regimen and dates
- Immunotherapy (binary; yes/no), with details of regimen and dates
- Did the participant decline the offer of any treatment (binary; yes/no) with details of what and why"
- Involvement with palliative care teams, including specialist interventions e.g. cordotomy, with dates

Imaging

- Baseline chest x-ray (binary; yes/no for common abnormalities, including pleural plaques, pleural effusion, pleural thickening, hydropneumothorax, other abnormality)
- Thoracic ultrasound
    - Size of effusion (categorical; costophrenic angle only, <25% of hemithorax, 25-50% of hemithorax; >50% of hemithorax)
    - Presence of loculations (categorical; free-flowing, mild, moderate or severely loculated, with pictorial guidance for each category)
- Baseline computer tomography (CT) scan (categorical; TNM stage)

Blood and pleural analysis

- Full blood count (continuous; SI units)
- Urea and electrolytes (continuous; SI units)
- Liver function tests (continuous; SI units)
- C-reactive protein (continuous; SI units)
- Lactose dehydrogenase (continuous; SI units)
- Serum mesothelin (continuous; SI units)
- Calculated neutrophil-to-lymphocyte ratio (continuous; numerical and binary; ≥4 or <4)
- Pleural fluid LDH, protein and glucose (continuous; SI units)

Brims prognostic score

Categorical (groups 1-4), calculated using predictive decision tree as described in Brims et al, 2016 (J Thorac Oncol. 2016;11(4):573-82).

Survival Analysis

	Crude mortality analysis			Adjusted mortality analysis		
	HR	95% CI	p	HR	95% CI	p
Age	1.03	1.01-1.06	0.02	1.03	0.99-1.07	0.13
Sex	0.91	0.54-1.53	0.71	0.74	0.34-1.63	0.46
Performance status >2	3.02	1.90-4.80	<0.001	3.96	2.14-7.30	<0.001
Non-epithelioid disease	1.71	1.06-2.75	0.03	1.83	1.01-3.31	0.045
Non-pleural disease	1.91	0.77-4.76	0.16	2.10	0.45-9.88	0.35
NLR	1.07	1.04-1.11	<0.001	1.07	1.03-1.11	0.001
Stage	1.36	1.11-1.66	0.003	1.13	0.98-1.31	0.08
NLR>4	1.69	1.13-2.52	0.011	Not included in multivariable model		
Discussion at regional MDT	0.84	0.48-1.47	0.54			
